# Two-photon polymerization enabled multi-layer liquid crystal phase modulator

**DOI:** 10.1038/s41598-017-16596-8

**Published:** 2017-11-24

**Authors:** Yun-Han Lee, Daniel Franklin, Fangwang Gou, Guigeng Liu, Fenglin Peng, Debashis Chanda, Shin-Tson Wu

**Affiliations:** 1 CREOL, The College of Optics and Photonics, University of Central Florida, Orlando, Florida 32816 USA; 2 Department of Physics, University of Central Florida, Orlando, Florida 32816 USA; 3 NanoScience Technology Center, University of Central Florida, Orlando, Florida 32826 USA

## Abstract

The performance of liquid crystal (LC) spatial light modulators depends critically on the amount of cumulative phase change. However, for regular phase modulators, a large phase change comes with a slow time response penalty. A multi-layer liquid crystal (LC) spatial light modulator offers a large phase change while keeping fast response time due to the decoupling between phase change and time response through engineered sub-micron scaffold. Here, we demonstrate specially designed 2- and 3-layer LC cells which can achieve 4 times and 7 times faster response time than that of conventional single-layer LC phase modulator of equivalent thickness, respectively. A versatile two-photon laser lithography is employed for LC cell scaffolding to accurately verify theoretical predictions with experimental measurements.

## Introduction

Liquid crystal (LC) phase modulators have become a standard class of instruments for the control of light. Through the field driven manipulation of their internal anisotropic medium, these devices can allow precise modification of phase, amplitude and polarization of incident beams. When scaled and implemented into addressable arrays, spatial phase modulators (SLMs)^1^ give near arbitrary control of wave fronts and are commonly used for wave-front control^2,3^, programmable femtosecond optical pulse shaping^4^, fiber-optic communication^5^, tunable-focus lens and gratings^6–8^ and quantum computation^9^. For these applications, SLMs should exhibit a large phase change (*δ* ≥ 2π), fast response time (*τ* ~ 1 ms), and a low operating voltage (≤15 V). However, these device attributes are intrinsically tied and optimization of one performance metric often leads to the detriment of others. For example, the phase change of a homogeneous cell is governed by the LC birefringence (Δ*n*), cell gap (*d*) and wavelength (*λ*) as:

1 $$\delta =2\pi d{\rm{\Delta }}n/\lambda .$$

On the other hand, the response time of the LC cell is determined by^10^:

2 $$\tau =\frac{{\gamma }_{1}}{{K}_{11}}\frac{{d}^{2}}{{\pi }^{2}},$$

where *γ*_1_ is the rotational viscosity and *K*_11_ is the splay elastic coefficient of the employed LC. From Eqs (1) and (2), a straightforward approach to obtain a large phase change is to increase the cell gap. However, this results in an increased response time. Several approaches have been investigated to simultaneously achieve large phase change and fast response times such as reflective liquid-crystal-on-silicon (LCoS)^11^, polymer network liquid crystals (PNLCs)^12–18^, and more recently blue phase liquid crystals (BPLCs)^19,20^. Each approach has its own pros and cons. For instance, the doubled path length of reflective LCoS offers twice the phase change compared to a transmissive modulator of the same LC layer thickness, but are generally more complicated and expensive to implement due to their nonlinear beam path. To enable a fast transmissive modulator, PNLCs decouple phase from response time through the additional anchoring force provided by UV-polymerized network. The nano-scaled domains within the network significantly accelerated the reorientation process and a sub-millisecond response was demonstrated^16^; however, this randomly-formed polymer web scatters visible light and shields applied fields resulting in relatively high operating voltages. For these reasons, a new approach is needed to achieve a large phase change while simultaneously keeping a fast response time.

In recent years, the maturity of nano-fabrication based on two-photon polymerization (TPP)^21,22^ has extended the horizon of ultra-fine devices. TPP utilizes pulsed infrared laser for polymerization. When focused, the transient high intensity induces two-photon absorption in precursors to form a sub-micron polymerized spot. By steering the focusing spot, hollow structure with nanoscale feature can be easily fabricated. This provides unique advantages for fabricating over traditional lithography methods and thus many previously difficult or impossible structures can now be realized^23–27^.

In this work, we demonstrate an engineered TPP phase modulator based on multi-layer structure to obtain large phase change and fast response time simultaneously. The structured layers separate the LC medium into well-defined sub-layers with nano-scale features. In such a multi-layer cell, the phase change is determined by the total LC thickness, while the response time is only determined by the thickness of each sub-layer. This allows the increment in total phase change while maintaining fast response time, without introducing scattering or hysteresis as in the case of PNLC. As a proof-of-concept, we fabricated a 2-layer and a 3-layer LC cells. We experimentally verified the response time improvement of 4 times and 7 times for the 2- and 3-layer structures, respectively, compared to the conventional single-layer structure with same phase modulation. The 4x response time enhancement factor for the 2-layer system exactly follows from the analytical prediction of Eq. (2). However, the improvement factor (7 times) for the 3-layer structure deviates from the expected 9 times improvement due to the unequal sub-layer gaps. The underlying physics is verified through device simulation and experimental measurements.

## Results

**Figure 1:**
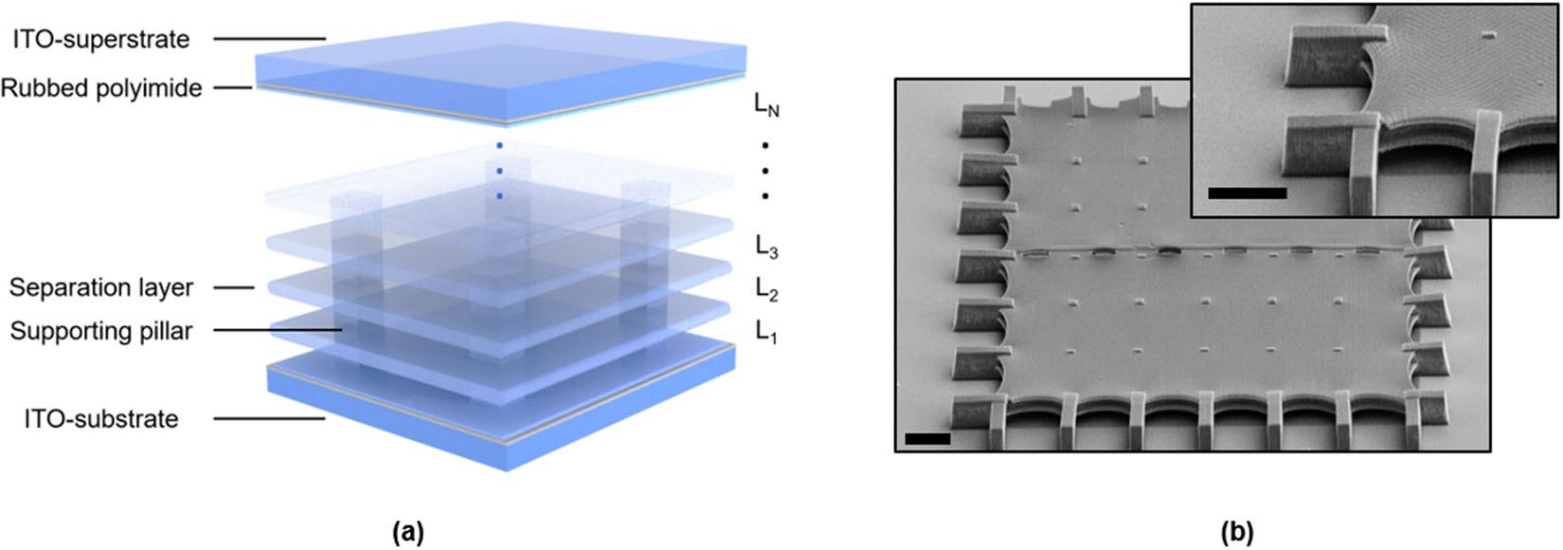
(**a**) Illustration of the multi-layer structure with N being the number of liquid crystal layers. (**b**) The SEM image of a three-layer LC cell. Scale bar: 10 *µ*m.

Figure 1(a) depicts the schematic of the multi-layer liquid crystal (MLC) modulator, where N refers to the number of liquid crystal layers that are supported by N−1 separation layers. The corresponding SEM image is shown for a sample with N = 3 (after covered with superstrate).

**Figure 2:**
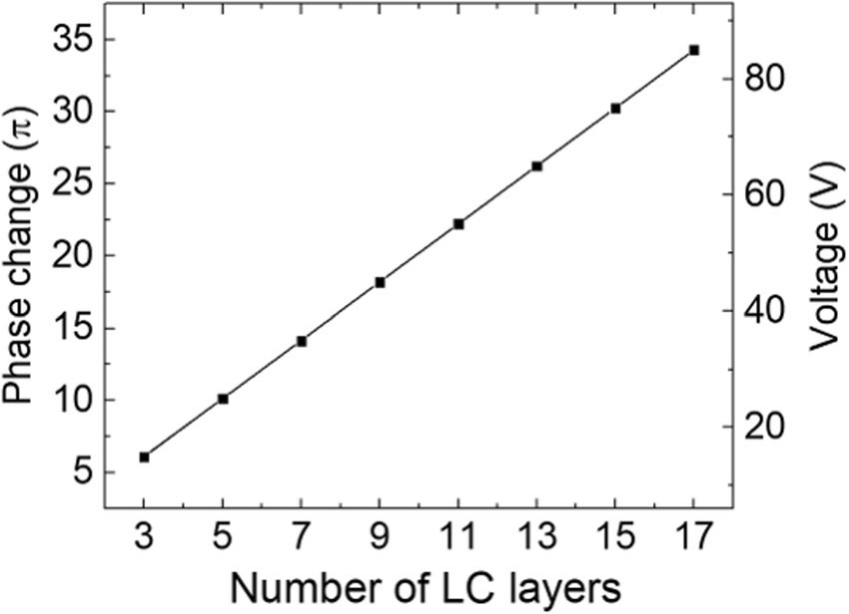
The simulated correlation between number of LC layers (N), driving voltage and phase change at 633 nm with each layer being 2.2 *µ*m. At N = 6, the phase change reaches 12.1 π at 30 V_rms_ for λ = 633 nm, which corresponds to δ = 2.0 π at λ = 3 *μ*m.

To understand the relation between the number of layers N, phase change *δ* (at *λ* = 633 nm) and driving voltage, we simulate a set of devices while maintaining a constant relaxation time (a constant sub-layer thickness of 2.2 *µ*m) using DIMOS liquid crystal simulator software, as shown in Fig. 2. Due to the independency of LC layers, a good linear relation holds. Note that with this configuration, increasing the number of layer and phase change does not slow down the response time of the whole device, as discussed before. At N = 6, the total phase change at 30 V_rms_ reaches 12.1 *π* for λ = 633 nm, which corresponds to *δ* = 2.0 *π* at λ = 3 *μ*m. To accommodate a longer wavelength, e.g. λ = 5 *μ*m, we should increase the layer number to 12.

It is noteworthy that in multi-layer approach, each layer is preferentially kept at the same thickness with the same anchoring force and uniform anchoring direction, and yet these parameters may be configured at will with the current TPP process for versatile multi-layer LC tunable devices not limiting to phase modulators.

**Figure 3:**
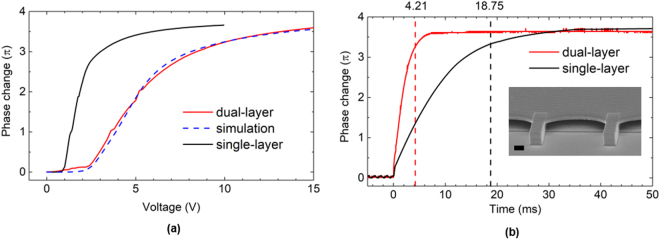
A dual-layer (N = 2) sample compared to a conventional single-layer sample with the same effective cell thickness of 3.4 µm. (**a**) The measured and simulated voltage dependent phase change of the single-layer and double-layer samples at λ = 633 nm. (**b**) The relaxation time of phase change from 0–90% for dual and single layer samples are 4.21 ms and 18.75 ms, respectively. Inset: the SEM image of a dual-layer sample, scale bar: 2 µm.

In Fig. 3, the experimental measurement with N = 2 sample (solid red curve) is compared with a conventional single-layer sample (solid black curve). The conventional single-layer sample had an effective cell gap of 3.4 *µ*m so that the total phase changes of the N = 2 sample and the single-layer sample are roughly the same. Figure 3(a) shows the measured and simulated voltage-dependent phase change of the two fabricated samples (N = 1 and 2). The blue dashed lines represent the simulation results, again obtained by the DIMOS software including the voltage shielding effect of the separation layers. The simulation and experimental results agree well. Also noticed from Fig. 3(a), the dual-layer sample exhibits a higher operation voltage than the single-layer one (black curve) for the following two reasons: 1) the additional anchoring force from the separation layer, which acts against molecular reorientation induced by the applied voltage, and 2) the voltage-shielding effect from the polymer separation layer. For the dual-layer sample, a phase change of 3.6 π was obtained at 633 nm at 15 V_rms_. Further, to ensure releasing from a saturated voltage, we measured the relaxation response upon releasing voltage from 20 V_rms_, as shown in Fig. 3(b). The dashed red lines (at 4.21 ms) and black lines (at 18.75 ms) mark the decay time for the 0–90% phase change. The improvement is slightly more than 4-fold, as predicted by Eq. (1). Note that in the conventional single-layer sample, a quick phase change occurred at near t = 0 due to the fast backflow near the alignment layers. This causes an overshoot of LC reorientation in the center, resulting in a slow backflow effect, which significantly increases the overall relaxation process^28,29^. The dual-layer sample greatly minimizes this backflow effect due to the additional anchoring surfaces and thinner cell gap for each sub-layer.

If such a dual-layer device is intended for longer wavelength say λ = 1.06 *µ*m, then the estimated phase change is about 1.86 π at 15 V_rms_ because of the increased wavelength and decreased Δ*n*. To obtain δ ≥ 2π, we can either increase the sub-layer thickness or build more layers. The former will undoubtedly increase the response time as explained above. Therefore, we further explore the three-layer geometry.

**Figure 4:**
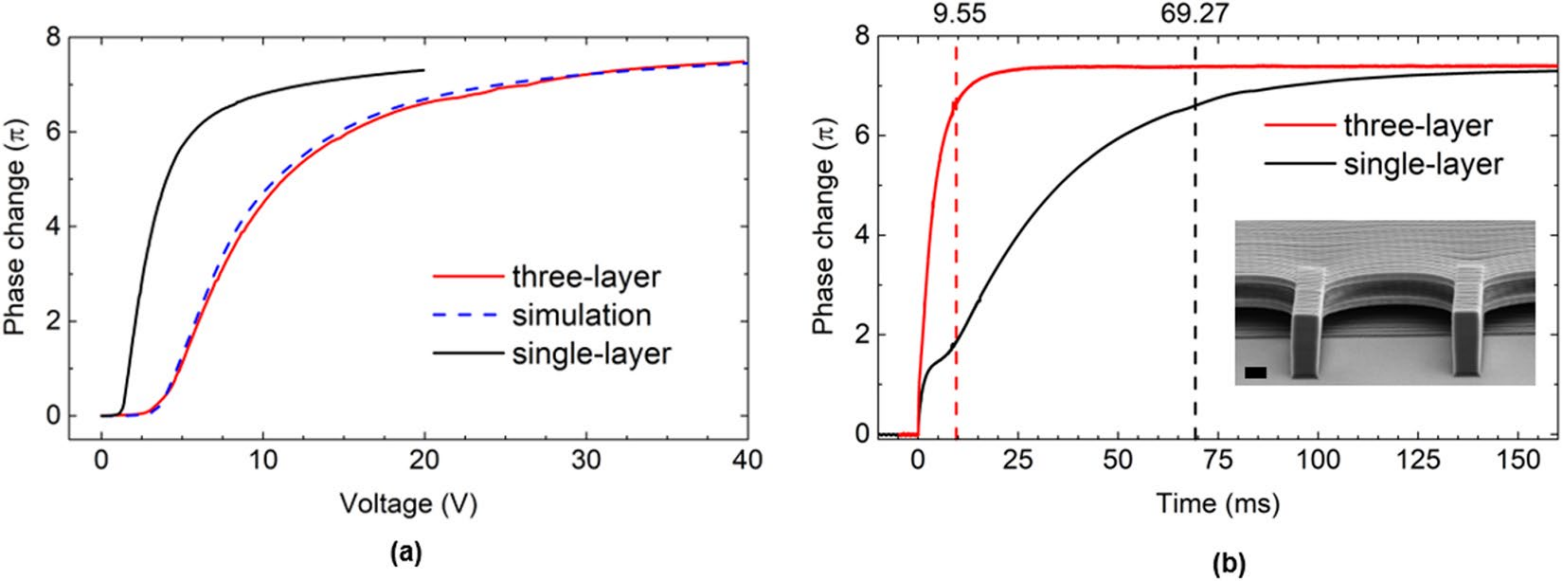
A three-layer (N = 3) sample compared to a conventional single-layer sample with the same effective cell thickness of 6.6 µm. (**a**) The voltage versus phase change shows a phase change of 7.2π at 30 V_rms_. λ = 633 nm. (**b**) The relaxation time of phase change from 0–90% for the three- and single-layer samples are 9.55 ms and 69.27 ms, respectively. Inset: the SEM image of a three-layer sample, scale bar: 2 µm.

Figure 4 depicts the simulated and measured results of our three-layer test cell. To achieve a larger phase change for short-wavelength infrared (SWIR) applications, we slightly increased the height of each sub-layer to 2.2 *µ*m so that the three-layer sample has an effective thickness of 6.6 *µ*m. Again, we compare this three-layer sample to a single-layer sample with the same effective cell gap. Figure 4(a) shows the voltage-dependent phase change of the three-layer sample compared to the single-layer sample. At 30 V_rms_, the phase change reaches 7.2 *π* for *λ* = 633 nm. This corresponds to *δ* = 3.7 *π* at 1.06 *µ*m and *δ* = 2.4 *π* at 1.55 *µ*m. Figure 4(b) shows the transient relaxation process when releasing from 30 V_rms_. The response time is reduced from 69.27 ms to 9.55 ms, showing a 7.2x improvement. Ideally, the response time should be 9-time faster as expected from Eq. (1). The main reason for the discrepancy is a slight deviation in individual sub-layer heights. As measured from SEM, the bottom layer thickness was 1.95 *µ*m and the second layer was 2.27 *µ*m. Thus, the top layer thickness was determined to be 2.38 *µ*m after subtracting the total thickness of the bottom two layers. As a result, the thickest layer (2.38 *µ*m) dominates the relaxation process. As estimated through Eq. (1), this corresponds to a 7.6x enhancement instead of 9x, which is consistent with the measured response time.

## Discussion

MLC allows the decoupling between response time and total phase change, and therefore allows a larger phase modulation at the same response time. This is critical for application in longer wavelength region. Currently, PNLC is the leading candidate for fast SWIR SLM. Due to the small average domain size, PNLC exhibits submillisecond response time at 1.06 *μ*m^30^. However, the major problems are high operation voltage (>80 V for a transmissive SLM), noticeable hysteresis, and double relaxation times. In the visible spectral region, PNLC scatters light strongly. TPP-enabled multi-layer LCs avoid these issues due to the well-defined polymer structure.

**Figure 5:**
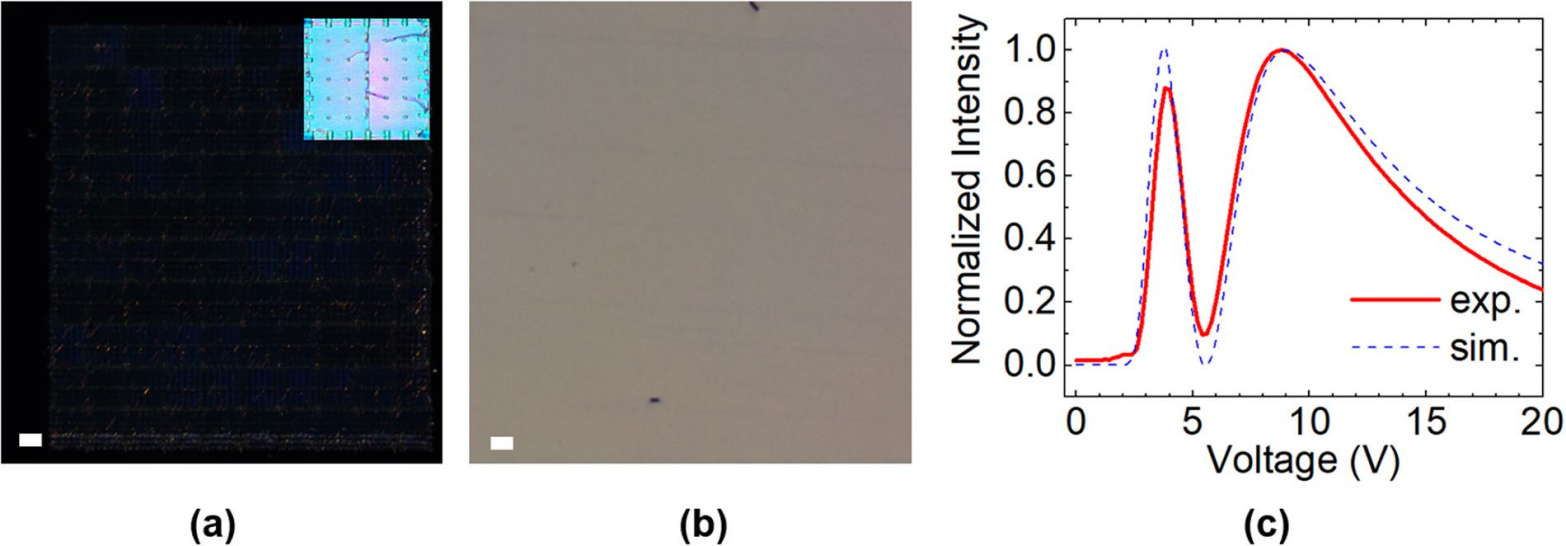
The dark-field micrographs of (**a**) a three-layer sample and (**b**) a PNLC sample at 40 V_rms_, showing a reduced scattering for MLC. (**c**) The voltage-transmittance curve (λ = 633 nm) of a two-layer MLC sample between crossed polarizers, showing the scattering-induced discrepancy between the experimental and simulation results. Scale bar: 50 *µ*m. Inset: the bright-field micrograph of the three-layer sample.

Figure 5 shows the dark-field micrographs of a three-layer structure (Fig. 5(a)) and a typical PNLC (Fig. 5(b), 7 wt% RM257 in a large Δε LC host HTG-135200), both were driven at 40 V_rms_. The samples were illuminated at 45° with a collimated white LED source. Even for regions near pillars and corners, our multi-layer structure only shows little scattering - much less than PNLC in which the whole region is bright due to scattering. In the inset of Fig. 5(a), the bright field image shows the major sources of the scattering in MLC are defect lines and degenerate alignment around the supporting pillars. The slight scattering of MLC manifests in the voltage-transmittance curve as shown in Fig. 5(c) for a two-layer MLC sample between crossed polarizers. The peak and valley does not reach simulated height and depth as a result of scattering. Such scattering can be further reduced by optimizing the device structure to suppress the defects in multi-layer structures.

**Figure 6:**
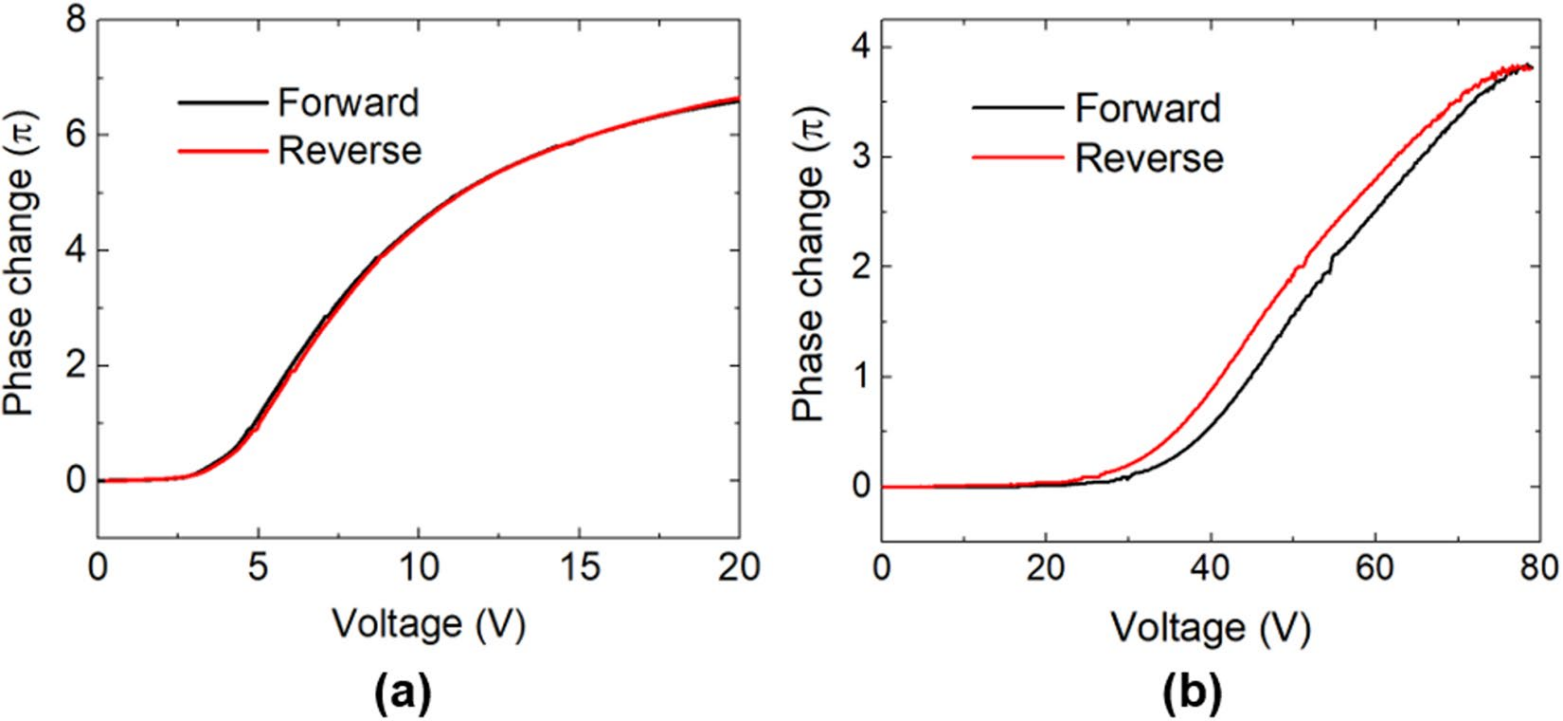
Hysteresis of (**a**) MLC and (**b**) PNLC. The discrepancy between forward and reverse processes shows visible hysteresis for a typical PNLC.

In Fig. 6, we investigate the hysteresis behavior of our multi-layer structure. In the forward scan, we applied a 1-kHz voltage from 0 to 20 V_rms_ for the three-layer sample, and 0 to 80 V_rms_ for the PNLC sample. In the backward scan, we lowered the voltage to 0 gradually. The process was performed with a dwell time of 50 ms and a step of 0.2 V. The solid polymer structure in the multi-layer sample (Fig. 6(a)) provides inherently hysteresis-free operation, while the PNLC sample (Fig. 6(b)) exhibits a 3% hysteresis, presumably due to the network distortion under applied voltage. It is also noteworthy that the accompanying double-relaxation issue found in PNLCs is not found in our multi-layer LC cells.

Currently, the writing time for a three-layer sample with a block size of 180 *µ*m by 180 *µ*m lies around 20 minutes. To scale up to a half-inch device, it will take a long time. However, this slow fabrication process can be improved in the future as the high power lasers with faster scanning system and multi-beam TPP are available.

## Conclusion

We have demonstrated, for the first time, a multi-layer LC phase modulator fabricated through two-photon polymerization. With a dual-layer structure, we observed a more than 4-fold improvement in response time with a doubling of the driving voltage. The three-layer LC sample exhibits 7-fold faster response and provides a phase change of 7.2 π at 30 V_rms_. While PNLCs provide sub-millisecond response time, we show that the multi-layer LCs allow negligible scattering, hysteresis-free and large phase-change at lower driving voltage, while maintaining a reasonably fast response time. The TPP-enabled multi-layer structure not only opens a new gate for spatial light modulators but also provides a nice flexibility allowing versatile design of controlled layer thickness and complex surface anchoring. We foresee versatile multi-layer LC devices incorporating polarization, focusing and phase modulation in near future.

## Methods

Figure 1(a) illustrates the multi-layer LC device structure. The polymer structure is formed on one side of an ITO-coated glass substrate through TPP in a commercial photoresist, IP-DIP (Nanoscribe GmbH). A 2D galvanometer scanner is utilized to steer the focal point of a 780 nm pulsed laser to expose lines of polymer connected side-by-side to form a uniform surface. The periodicity and direction of the written lines creates nanoscale grooves in the surface, which provides a controllable anchoring for the LC molecules in both direction and anchoring force^31,32^. These separation layers were formed to define cavities L1 through LN, which will later be infiltrated with liquid crystal. We define N as the number of liquid crystal layers. In this experiment, these layers have nominal thickness of 1.7 *μ*m for N = 2 and 2.2 *μ*m for N = 3. The separation layers have a nominal thickness of 800 nm. Supporting pillars were formed *in situ* with a width of 1 *μ*m. After TPP, the sample was gently placed in 1,2-Propanediol monomethyl ether acetate (PGMEA) solution for 20 min to remove the unexposed photoresist, and then it was placed in isopropyl alcohol (IPA) to remove PGMEA. The sample was then vertically held at 20 cm above a 200 °C hot plate for 2 min to evaporate the solvent.

Figure 1(b) shows the SEM image of the sample with N = 3. The total area was 180 by 180 *µ*m^2^. The pillar distance was set to be 15 *µ*m, and the pillars at boundaries were elongated to ensure the structure does not peel off during evaporation process. Stitching outlets were also formed in the middle to facilitate the developing process. Clear vacancies between the separation layer and the substrate can be observed. A thin film of polymer (<100 nm) with grooves was formed on the substrate to serve as an alignment layer. The same horizontal grooves can be seen on the separation layers, and it was found that the boundary shrank slightly after evaporation, forming a slightly curved feature.

To complete the LC cell, the TPP polymer substrate was UV cured onto an ITO-superstrate coated with rubbed polyimide to provide homogeneous alignment. The distance between these two substrates were controlled with silica spacers at 4.3 *µ*m and 8.45 *µ*m to create effective cell gaps (gap spacing excluding the polymer separation layer thickness) of 3.4 *µ*m and 6.6 *µ*m for the N = 2 and N = 3 samples, respectively. The cell was then filled with a liquid crystal mixture LCM-1660 (LC Matter, USA) to achieve large phase change due to its high birefringence (Δn = 0.38 at λ = 632.8 nm).

To measure the phase change, we oriented the test cell at 45° between crossed polarizers, and applied an AC (1 kHz, square wave) voltage. We focused a He-Ne laser (λ = 633 nm) beam onto the active area, and the transmitted light after the analyzer was received by a photodiode detector. This way, the phase change at a given voltage, *δ*(*V*), can be calculated by the transmittance (*T*) as:

3 $$T={T}_{o}\,{\sin }^{2}\frac{\delta (V)}{2},$$

where *T*_o_ refers to the maximum transmittance.
